# Optimized Thermoelectric Properties of Sulfide Compound Bi_2_SeS_2_ by Iodine Doping

**DOI:** 10.3390/nano12142434

**Published:** 2022-07-15

**Authors:** Chongbin Liang, Bushra Jabar, Chen Liu, Yuexing Chen, Zhuanghao Zheng, Ping Fan, Fu Li

**Affiliations:** Shenzhen Key Laboratory of Advanced Thin Films and Applications, College of Physics and Optoelectronic Engineering, Shenzhen University, Shenzhen 518060, China; 2060451017@email.szu.edu.cn (C.L.); bushrajabbar_786@outlook.com (B.J.); 2070456054@email.szu.edu.cn (C.L.); chenyx@szu.edu.cn (Y.C.); zhengzh@szu.edu.cn (Z.Z.); fanping@szu.edu.cn (P.F.)

**Keywords:** thermoelectric material, Bi_2_SeS_2_, doping, thermoelectric property

## Abstract

The Te-free compound Bi_2_SeS_2_ is considered as a potential thermoelectric material with less environmentally hazardous composition. Herein, the effect of iodine (I) substitution on its thermoelectric transport properties was studied. The electrical conductivity was enhanced due to the increased carrier concentration caused by the carrier provided defect *I_se_*. Thus, an enhanced power factor over 690 μWm^−1^K^−2^ was obtained at 300 K by combining a moderate Seebeck coefficient above 150 µV/K due to its large effective mass, which indicated iodine was an effective n-type dopant for Bi_2_SeS_2_. Furthermore, a large drop in the lattice thermal conductivity was observed due to the enhanced phonon scattering caused by nanoprecipitates, which resulted in a low total thermal conductivity (<0.95 Wm^−1^K^−1^) for all doped samples. Consequently, a maximum *ZT* value of 0.56 was achieved at 773 K for a Bi_2_Se_1−x_I_x_S_2_ (x = 1.1%) sample, a nearly threefold improvement compared to the undoped sample.

## 1. Introduction

Thermoelectric (TE) materials directly convert electrical and thermal energy without emitting harmful substances and are considered environmentally friendly materials with huge development potential and broadly applicability [1,2,3,4]. Generally, the energy conversion efficiencies of TE materials are evaluated by *ZT* (*ZT = σS^2^T/κ*), where *σ*, *S*, *κ,* and *T* are the electrical conductivity, Seebeck coefficient, thermal conductivity, and the absolute temperature, respectively [5,6,7]. Therefore, an outstanding TE material should have a high *ZT* with high electrical conductivity, large Seebeck coefficient, and low thermal conductivity. The optimization of these intuitive interdependent parameters poses a significant challenge. However, the design of rational experimental strategies has continuously rewritten the record of *ZT* over the past twenty years [8,9,10,11,12,13].

As two important conventional TE materials, Bi_2_Te_3_ and PbTe are the best and most stable materials used near room temperature and mid-temperature [14,15]; however, large-scale commercial applications in the future are limited due to the extensive use of toxic Pb and Te [16]. Thus, developing Te-free and Pb-free candidates with high TE performance represents a significant and important challenge [17,18]. Recently, environmentally friendly sulfide compounds have received widespread attention. Bi_2_S_3_ is a typical example of the sulfide counterpart of Bi_2_Te_3_, with a similar crystal structure [19,20]. Despite its poor electrical conductivity and high thermal conductivity that results in a dismally low *ZT* (0.055), TE properties have been optimized using several effective strategies, which include elemental doping, texturing, and microstructure design [21,22,23,24,25,26]. However, most reported *ZT* values are < 0.6, relatively low compared to traditional materials [21,22,23,24,25,26]. Poor TE properties for the sulfide compounds stem primarily from low electrical conductivity and high lattice thermal conductivity due to the light atomic weights and either too high or too low carrier concentrations.

Recent reports revealed that the ternary sulfide compound Bi_2_SeS_2_ has TE potential with a high *ZT* (>1.0) [27,28]. Its crystalline structure was similar to Bi_2_S_3_. But the Bi_2_SeS_2_ band gap of ~1.0 eV was narrower than Bi_2_S_3_ (~1.35 eV), which caused reasonably satisfactory electrical conductivity. Combined with low thermal conductivity (<0.8 W m^−1^K^−1^), a *ZT* value of 0.25 was obtained for the pure sample [29]. By tuning the electrical conductivity via Cu doping and suppressing the lattice thermal conductivity by forming nanoprecipitates, the *ZT* value increased to 0.7 at 723 K, and to 1.0 at 773 K [27,30]. Recently, Br served as an effective carrier donor and induced a partial Bi_2_SeS_2_ phase change from *Pnma* to *Pnnm*, which formed a quasi homo-composition and a hetero-structure (*ho*C-*he*S) nanocomposite [28]. This caused a significant lattice thermal conductivity decrease and enhanced power factor, which resulted in a large *ZT* value improvement.

Halogens are well-known n-type dopants for the Bi_2_X_3_ family (X = Te, Se, and S), with defects in Cl_x_, Br_x_, or I_x_ providing free electrons to increase carrier concentrations [31,32]. Aside from Br doping, the effect of single Cl or I doping on TE transport properties of Bi_2_SeS_2_ has not been reported. Whether Cl or I would induce the phase transition of Bi_2_SeS_2_ merits consideration. Hence, this motivated us to conduct this study. Herein, iodine (I) was chosen as a dopant in the Bi_2_SeS_2_ system. Although no phase transition was found after iodine doping, an enhanced power factor due to the increased carrier density, together with a reduced lattice thermal conductivity due to the enhanced phonon scattering, was obtained. A maximal *ZT* of 0.56 at 773 K was achieved for Bi_2_Se_1−x_I_x_S_2_ with x = 1.1%, three times higher than the undoped sample, which indicated iodine was an effective n-type dopant for Bi_2_SeS_2_.

## 2. Experimental

A series of Bi_2_Se_1−x_I_x_S_2_ (x = 0, 0.1%, 0.3%, 0.5%, 0.7%, 0.9%, 1.1%, and 1.3%) ingots were synthesized by a combination of solid-state reaction and spark plasma sintering (SPS) using high-purity powders of Bi (99.99%), Se (99.99%), S (99.95%) and BiI_3_ (99.99%). Stoichiometric powder mixtures were roughly mixed and placed into glass ampoules according to their compositions. The ampoules were sealed under vacuum (<10^−3^ Pa) and heated at 1173 K for 12 h with subsequent annealing at 773 K for 48 h. Ingots were obtained upon cooling to 300 K and pulverized into micron-sized powders by hand grinding. The powder was placed into a mold and sintered in as SPS furnace at 773 K for 5 min with an axial pressure of 50 MPa to form column-shaped bulk sample. Two pieces were obtained from one bulk sample. One piece was used to measure the electrical conductivity and Seebeck coefficient along the direction perpendicular to the SPS pressing direction; the other piece was used to test its thermal conductivity in the same direction.

Crystal structures were determined by X-ray diffraction (XRD, Ultima IV, Riguku, Japan) using Cu Kα radiation and a scanning rate of 5°/min. Rietveld refinement was performed using the Generalized Structural Analysis System (GSAS-II) program. The micromorphologies of the fractured surfaces and backscattered electron imaging (BES) on the polished surfaces were detected via field scanning electron microscopy (FSEM, Supra 55 Sapphire, Zeiss, Germany). The energy dispersive spectroscopy (EDS) equipment attached to the FSEM analyzed the elemental compositions and distributions. The electrical resistivity and the Seebeck coefficient were measured from room temperature to 773 K using an electric resistance/Seebeck coefficient measuring system (ZEM-3, Ulvac-Riko, Japan). The Hall coefficients (*R_H_*) at room temperature were tested by a Van der Pauw technique. Based on the expression *μ = σR_H_* and *n* = 1/(*eR_H_*), where *σ* is the electrical conductivity, the carrier mobility (*μ*) and the carrier concentration (*n*) were calculated. The thermal diffusivity (*D*) was determined using laser flash diffusivity (Netzsch LFA467, Germany), which was then used to calculate the total thermal conductivity (*κ*) using *κ = DC_p_ρ,* where *ρ* is the bulk density of the sample, and *C_p_* is the heat capacity. *C_p_* values were tested using two differential scanning calorimeters (DSC); a DSC200-F3 yielded *C_p_* values from 298–523 K; a DSC404-C gave *C_p_* values from 523–773 K. Densities were determined by the Archimedes method.

## 3. Results and Discussion

**Figure 1:**
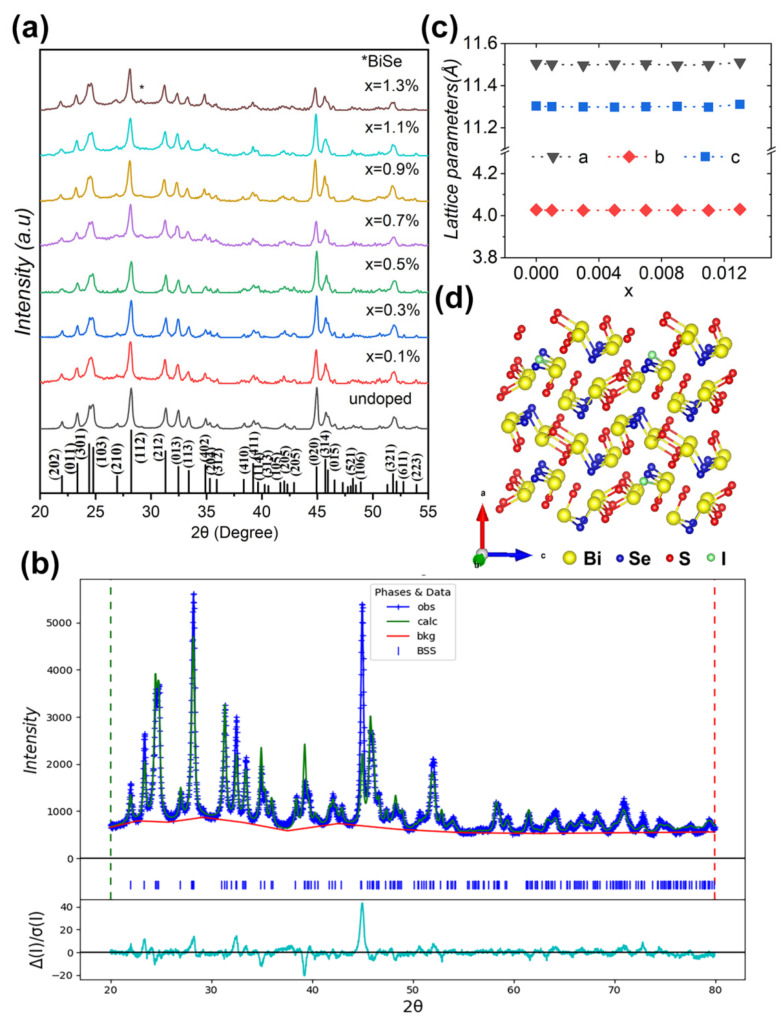
(**a**) XRD patterns for Bi_2_Se_1−x_I_x_S_2_ (x = 0–1.3%). The asterisk represents the impurity peak of BiSe. (**b**) Rietveld refinement analysis from XRD patterns of Bi_2_SeS_2_, where obs represents observed, calc is calculated, bkg is background, and diff is the residual difference between the curves. (**c**) Variation of lattice parameters with the doped amount x. (**d**) Crystal structure along the *a*-axis for the iodine doped Bi_2_SeS_2_.

The XRD patterns for pristine and doped Bi_2_Se_1−x_I_x_S_2_ (x = 0–1.3%) samples are presented in Figure 1a. The main peaks agree with the standard XRD peaks of Bi_2_SeS_2_ polycrystalline, simulated from the atomic site occupation and lattice parameters a = 11.5044 Å, b = 4.0254 Å, and c = 11.2959 Å, as reported previously [19]. Reitveld refinement studied the doping-induced structural changes in the Bi_2_SeS_2_ system further (representative result is shown in Figure 1b). The XRD peaks of all samples match well with an orthorhombic *Pnma* phase. No phase transition can be found as the reported Br doped samples [28]. Figure 1c shows the I dopant-induced lattice parameter evolution, which was derived from the Reitveld refinement. According to the calculated formation energy [28], I preferentially occupies the Se site when doping in Bi_2_SeS_2_. The ionic radius of I^−^ (2.2 Å) exceeds Se^2−^ (1.98 Å), which should enlarge the lattice parameters. However, no obvious changes were observed for the three axes (Figure 1c), although slight increase in the *a*- and *c*-axes appears at high doping levels. One possible reason might stem from the low doping levels of I, which cannot cause these changes. Another reason might be ascribed to the volatilization of S, which may offset lattice parameters increases caused by I doping. In addition, a weak impurity peak appeared at ~29.21° in all samples, which corresponded to the BiSe (PDF#42-1045) phase as BiSe has a characteristic peak around this position. This impurity was commonly observed in previous reports for Bi_2_SeS_2_ systems and is thought to spontaneously form due to thermodynamic stability similar to Bi_2_SeS_2_ [29].

**Figure 2:**
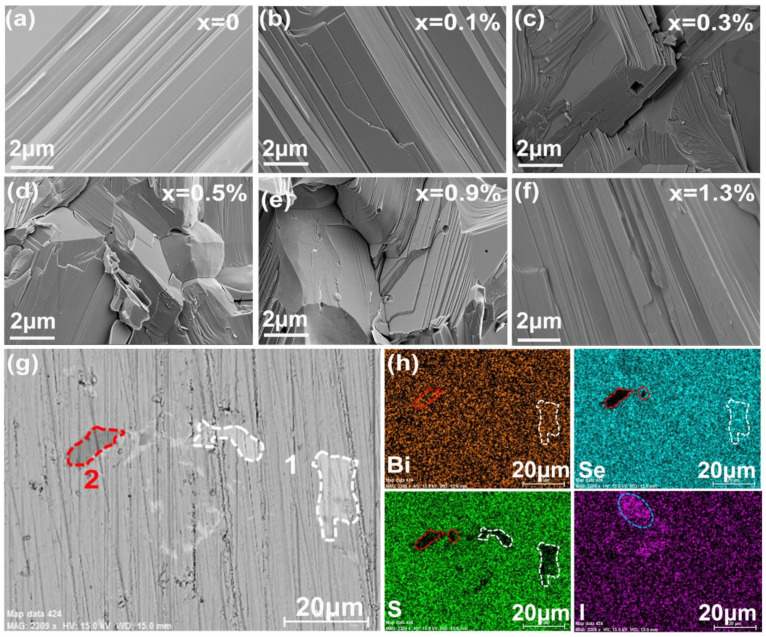
(**a**–**f**) SEM pictures of fresh fracture surfaces for Bi_2_Se_1−x_I_x_S_2_ with x = 0, 0.1%, 0.3%, 0.5%, 0.9%, and 1.3%; (**g**) BES image for Bi_2_Se_1−x_I_x_S_2_ (x = 1.3%). The lighter contrast areas are indicated as 1, while the deep contrast areas are indicated as 2; (**h**) Distribution of the corresponding elements Bi, Se, S and I on the sample surface in (**g**).

SEM pictures for the freshly fractured surface perpendicular to the press direction at different x doping levels are shown in Figure 2a–f. All samples show very dense microstructures, which resulted in a relatively high average density of 7.07 gcm^−3^ and very close to the theoretical value (7.12 gcm^−3^). Grain sizes in the tens of microns range for the undoped sample dropped to 2–5 μm when doped with I, which indicated that iodine inhibited the diffusion of the elements during sintering and prevented the grain growth. The grains are in a lamellar structure and an apparent orientation arrangement was seen in some areas, which suggested a preferential orientation. The composition and the distribution of Bi, S, Se, and I were detected by EDS on the polished surface. In a few areas, a sulfur deficiency was observed as shown in Figure 2h, and associated with the lighter contrast area indicated as [1] in the BES image of Figure 2g, which confirmed that BiSe precipitates formed in the matrix of Bi_2_Se_1−x_I_x_S_2_ (x = 0–1.3%) as mentioned in the XRD results. Iodine was homogeneously distributed in lower doped samples (x < 1.1%), but some areas of iodine enrichment were observed at elevated dopant levels (1.1% and 1.3%, Figure 2h), which indicated that some I did not enter the lattice and x = 1.1% could represent the iodine doping limit in Bi_2_SeS_2_. Furthermore, a corresponding enrichment of Bi appeared in very few areas for those two samples indicated as [2] in Figure 2h. This might be due to lower Se levels with increasing x in Bi_2_Se_1−x_I_x_S_2_, and decreased sulfur due to the volatilization, causing excess Bi to precipitate. No Bi or I nanoprecipitate peaks were observed in the XRD patterns, mainly because of their extremely low content.

**Figure 3:**
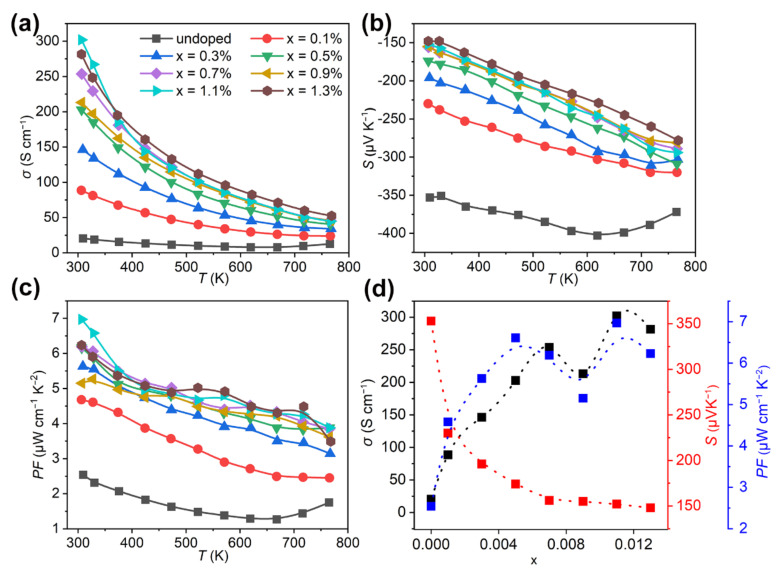
Temperature dependence of (**a**) electrical conductivity (σ), (**b**) Seebeck coefficient (*S*) and (**c**) power factor (*PF*) for Bi_2_Se_1−x_I_x_S_2_ (x = 0–1.3%). (**d**) Change of electrical transport parameters (σ, *S* and *PF*) with doping level at room temperature.

The temperature dependent electrical conductivity (*σ*), Seebeck coefficient (*S*), and power factor (*PF*) for Bi_2_Se_1−x_I_x_S_2_ (x = 0–1.3%) are shown in Figure 3a–c. Figure 3d presents the lattice parameters variation as a function of doping amount at room temperature. The pristine Bi_2_SeS_2_ sample has a poor *σ* (20.4 Scm^−1^) due to its low carrier concentration (*n*) (Figure 3a) and moderate *S* (350–400 μVK^−1^) (Figure 3b), which resulted in a low *PF* (<2.5 μWm^−1^K^−2^) as shown in Figure 3c. However, an obviously enhanced *σ*, up to 100 Scm^−1^, at 300 K was obtained for Bi_2_Se_1−x_I_x_S_2_ (x = 0.1%), five times higher than the undoped one. The *σ* roughly increases with increased doping levels (Figure 3d). For instance, increasing the doping levels from 0.3%, 0.5%, and 0.7% improved the *σ* from 146.3, 202.8 and 253.7 Scm^−1^ (Figure 3d), respectively. This value maximized at 302 Scm^−1^ for the 1.1% sample, although it dropped slightly for Bi_2_Se_1−x_I_x_S_2_ (x = 0.9%). Increasing x to 1.3% lowered the *σ* at room temperature. But from 300–773 K, the *σ* of Bi_2_Se_1−x_I_x_S_2_ (x = 1.3%) was better than the others (Figure 3a). As for the temperature-dependent *σ*, the *σ* for all doped samples decreased monotonically up to 773 K, which indicated a degenerate semiconductor. However, all data from doped samples exceeded the pristine sample.

**Figure 4:**
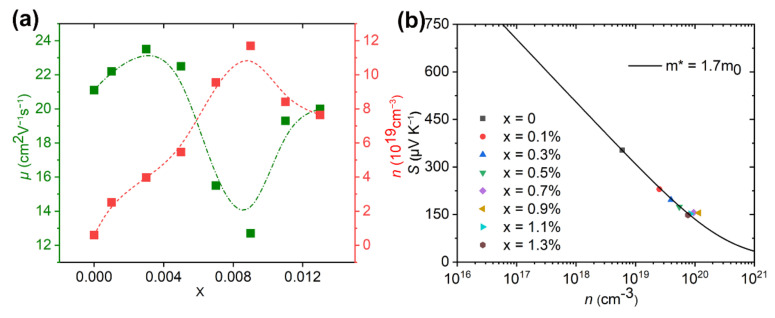
(**a**) Change of carrier concentration (*n*) and carrier mobility (*μ*) with doping content x, (**b**) Change of Seebeck coefficient (*S*) with carrier concentration (*n*). The line in (**b**) was calculated by the SPB model discussed in detail elsewhere [28,33]. m* is the electron effective mass at the Fermi level.

The *σ* was determined by the carrier concentration (*n*) and carrier mobility (*μ*) based on the expression of σ = *neμ*, where *e* represents the electron charge. Figure 4a shows *n* and *μ* as a function of the doping level at room temperature. The pristine sample has a low *n* of 5.97 × 10^18^ cm^−3^ caused by S vacancies as follows.

(1) $${\mathrm{Bi}}_{2}{\mathrm{SeS}}_{2}\;\to \mathrm{xS}↑+{\mathrm{xV}}_{S}^{2+}+2{\mathrm{xe}}^{-}$$

The upward arrow indicates sulfur volatilization. $V_{S}^{2+}$ represents an S vacancy. $e^{-}$ represents an electron. The *x* in Equation (1) represents the possible amount of S volatilization for the undoped sample. After doping with a small amount of I (x = 0.1%), σ increased to 2.52 × 10^19^ cm^−3^. With further increases of the doped content x to 0.3%, 0.5% and 0.7%, the *n* gradually increased to 3.97 × 10^19^ cm^−3^, 5.47 × 10^19^ cm^−3^ and 9.55 × 10^19^ cm^−3^, and maximized at 11.7 × 10^19^ cm^−^^3^ when the doped content was 0.9%. This enhancement should be caused by the carrier provider of *I*_se_, according to the following defect chemistry reaction.

(2) $${\mathrm{Bi}}_{2}{\mathrm{Se}}_{1-x}S_{2}I_{x}\to {\mathrm{xI}}_{\mathrm{Se}}^{+}+{\mathrm{xe}}^{-}$$

The $I_{\mathrm{Se}}^{+}$ indicates the replacement of Se by I in the lattice. More electron carriers were supplied if the iodine replaced Se in Bi_2_SeS_2_, which indicated I was an efficient dopant. This also confirmed that most iodine entered the lattice although some volatilized during sintering. Further increasing the doping levels to x = 1.1% and 1.3% caused the *n* to decrease to 8.41 × 10^19^ and 7.65 × 10^19^ cm^−3^. As mentioned above, I doping reaches the doping limit at x = 1.1%. In this situation, excessive levels of I can form nanoprecipitates as secondary phases. The interfaces between the secondary phases can significantly filter or block the electron carriers, which lowered *n*. The *μ* changes with increasing doping levels as shown in Figure 4a. Firstly, the value of *μ* slightly increases from 21.1 cm^2^V^−1^s^−1^ for the pristine sample to 22.2 cm^2^V^−1^s^−1^ and 23.5 cm^2^V^−1^s^−1^ when the sample doped with small amounts of x = 0.1% and 0.3%. It then decreases to 22.5, 15.5 and 12.7 cm^2^V^−1^s^−1^ with further increasing of the doped content x to 0.5%, 0.7% and 0.9%. Since *n* improved after iodine doping, the carrier-carrier scattering might be one of the scattering mechanisms causing the change of *μ*. In addition, the lattice imperfections introduced by I doping, impurity phases, and their phase boundaries could further scatter the carriers, which also play an important role in the carrier scattering. For samples doped with x = 1.1% and 1.3%, *μ* increases to 19.2 and 20 cm^2^V^−1^s^−^^1^ due to reduced carrier scattering, since the iodine doping reaches its limit as mentioned above. Moreover, grain sizes for samples doped with x = 1.1% and 1.3% increased as compared to samples with lower doped levels as shown in Figure 2, which also reduces carrier scattering because of the decreased grain boundary concentration. Combined with the *n* data, the increase of *σ* for the doped samples primarily resulted from the increase of *n*, while the reduced data for the doped sample with x = 0.9% was attributed to the reduced *μ*. Moreover, the improved *σ* and *n* for samples doped with x < 1.1% indicated that electron doping (I) successfully occurred in the Bi_2_SeSe_2_ system.

Figure 3b shows the Seebeck coefficient (*S*) for Bi_2_Se_1−x_I_x_S_2_ (x = 0–1.3%). The negative data over the entire temperature range suggested that Bi_2_SeS_2_ is an n-type semiconductor. Overall, the *S* for all doped samples improved with a temperature increase. The pristine Bi_2_SeS_2_ sample shows a relatively high *S* ~350 μVK^−1^ at room temperature. After iodine doping, the value dropped due to the enhanced carrier concentration. It gradually dropped to 148 μVK^−1^ as the doping level increased to 1.3%. According to the Mott formula [34], *S* can be expressed by the following equation:

(3) $$S=\frac{8\pi k_{B}^{2}T}{3eh^{2}}m^{*}{\left(\frac{\pi }{3n}\right)}^{\frac{2}{3}}\;\;\;\;\;\;\;\;$$

where *k_B_*, *h*, *e*, m* and *n* are the Boltzmann constant, the Planck constant, the electronic charge, the electron effective mass at the Fermi level, and the carrier concentration, respectively. This means increasing *n* would decrease *S*. In addition, an increase of m* should increase the *S*. To present a quantitative understanding of the microscopic carrier variations, densities of state effective mass m* are derived. Assuming that electron conduction occurs within a single parabolic band (SPB) with scattering dominated by the acoustic phonon model, the change of *S* with *n* can be plotted using the equations described elsewhere [33,35,36,37]. Herein, the relationship between *S* with the *n* is called the Pisarenko relation [33]. The solid curve of the SPB model fitted for the *S*(*n*) function is shown in Figure 4b. The Pisarenko relationship fitted well with an m* of 1.7*m^o^*, where *m^o^* is the electron mass, and indicates the band structure was not changed by I substitution. Herein, the m* of Bi_2_SeS_2_ was larger than Bi_2_S_3_ (~1.5*m^o^*) [38], which seems the key factor leading to the higher *S* but lower *μ* of Bi_2_SeS_2_.

Figure 3c gives the power factor (*PF*). Since the enhanced *σ* for the doped samples completely compensated for the *S* decline, the resultant *PF* enhanced significantly. A maximum *PF* of 697 μWm^−1^K^−2^ was achieved for Bi_2_Se_1−x_I_x_S_2_ (x = 1.1%) at 300 K, three times higher than the undoped sample, and larger than other reported data of Bi_2_SeS_2_ + CuI (<500 μWm^−1^K^−2^ at 300 K), Bi_2_S_3_ (<100 μWm^−1^K^−2^ at 300 K) and Bi_2_Se_3_ (<200 μWm^−1^K^−2^ at 300 K) [19,20,39].

**Figure 5:**
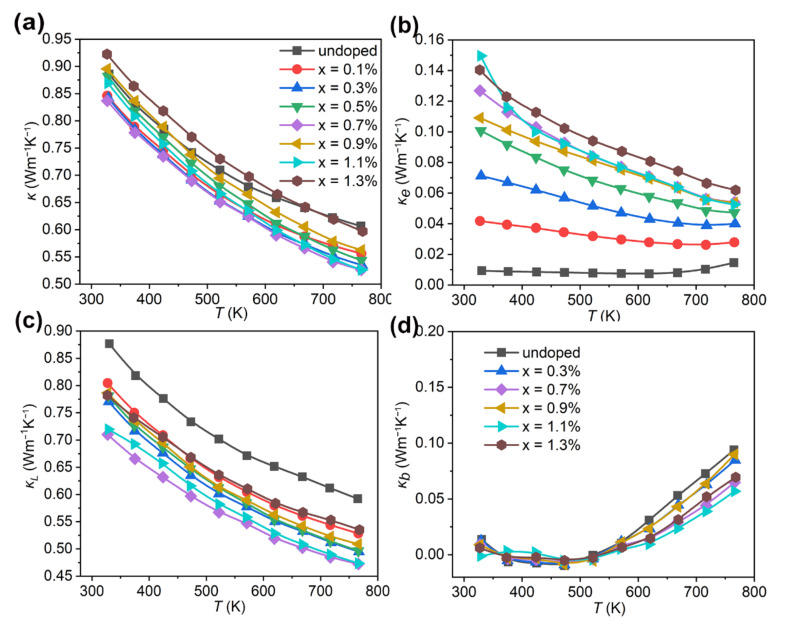
Thermal transport properties for Bi_2_Se_1−x_I_x_S_2_ (x = 0–1.3%). (**a**) Total thermal conductivity (*κ*), (**b**) electronic thermal conductivity (*κ_e_*), (**c**) lattice thermal conductivity (*κ_L_*), (**d**) bipolar thermal conductivity *(κ_b_*).

Figure 5a shows the total thermal conductivity (*κ*) for Bi_2_Se_1−x_I_x_S_2_ (x = 0–1.3%). The pristine Bi_2_SeS_2_ shows a low *κ* of 0.88 Wm^−1^K^−1^ at 300 K, which decreased further to 0.60 Wm^−1^K^−1^ at 773 K. This intrinsically low *κ* of Bi_2_SeS_2_ was due to its layered structure with weak bonding strength, because it acts as a boundary for effective phonon scattering sources in the Bi_2_SeS_2_ matrix [40]. The *κ* of the doped samples Bi_2_Se_1−x_I_x_S_2_ with x = 0.1%, 0.3%, 0.5%, 0.7%, 0.9% and 1.1% were all lower compared to the undoped sample. The lowest *κ* values of 0.83 Wm^−1^K^−1^ at room temperature and 0.52 Wm^−1^K^−1^ at 773 K were obtained for Bi_2_Se_1−x_I_x_S_2_ (x = 0.7%). Due to the high electronic thermal conductivity (*κ_e_*), discussed below, the *κ* of Bi_2_Se_1−x_I_x_S_2_ (x = 1.3%) was slightly higher than the undoped sample.

Generally, the *κ* is the sum of the lattice thermal conductivity (*κ_L_*) and the electronic thermal conductivity (*κ_e_*), depicted below:

(4) $$\kappa =\kappa {\;}_{e}+\kappa_{L}\;$$

The *κ_e_* was obtained through the Wiedemann–Franz relationship, *κ_e_ = LσT*, where *σ*, *T*, and *L* are the electrical conductivity, temperature, and the Lorenz number, respectively. Here, the *L* ranges from 1.50 × 10^−8^ to 1.80 × 10^−8^ ΩWK^−2^, which is calculated by applying the reduced Fermi energy *η*. The *κ_e_* for Bi_2_Se_1−x_I_x_S_2_ (x = 0–1.3%) is presented in Figure 5b. After doping with I, the *κ_e_* increased due to the increased *σ*. It gradually improved with higher dopant levels. However, the *κ_e_* holds only 5~17% of the *κ* for all doped samples, indicating that the *κ* is not predominated by *κ_e_*. The temperature dependence of *κ*_L_ is shown in Figure 5c. *κ*_L_ decreased significantly over the entire temperature range after doping. At room temperature, this parameter dropped from 0.87 Wm^−1^K^−1^ for the pristine sample to 0.78 Wm^−1^K^−1^ for Bi_2_Se_1−x_I_x_S_2_ (x = 0.3%), to 0.77 Wm^−1^K^−1^ for Bi_2_Se_1−x_I_x_S_2_ (x = 0.5%), and further to 0.71 Wm^−1^K^−1^ for Bi_2_Se_1−x_I_x_S_2_ (x = 0.7%). The significant *κ*_L_ reduction should result from the enhanced phonon scattering from the secondary phase of BiSe and the heavier relative atomic mass of I because it introduces strong phonon scattering due to the mass fluctuations. In addition, the increased grain boundary concentration caused by the reduced grain size after doping (Figure 2) also played an important role in phonon scattering. Further increasing the dopant to 0.9%, 1.1% and 1.3%, the *κ*_L_ increased slightly compared with other doped samples. One possible reason may be due to the redundancy of Bi-rich nano-precipitates with a relatively higher *κ*_L_ in the sample. Another possible reason may be the reduced grain boundary scattering due to the reduced grain boundary concentration and the increased grain size for high-doped samples. As shown in Figure 2, the grain size for the undoped sample decreased with iodine doping. However, with upping the dopant level to 0.9%, the grain size increased. The Bi-rich nano-precipitates might act as a sintering aid, contributing to the sample grain growth [41]. As for the data dependence of the temperature, the *κ_L_* for all samples decreased with increasing temperature. However, above 600 K, the value decreases slowly. This should be attributed to the bipolar effect. In fact, for a wide-gap semiconductor at elevated temperatures or narrow-gap semiconductor at room temperature, both holes and electrons contribute to transport. This indicates that bipolar thermal conductivity (*κ_b_*) should be considered. The *κ_b_* was separated from *κ* to clarify the contribution of *κ*_b_ at high temperatures, according to the method reported by Kitagawa et al. [42]. Figure 5d shows the *κ*_b_ for some representative samples. It shows that *κ*_b_ has been reduced for the doped samples. It dropped from 0.094 Wm^−1^K^−1^ to 0.056 Wm^−1^K^−1^ for doped levels from x = 0 to x = 1.1% at 773 K, likely due to the trapping of minority carriers within the dense nanoscale regions [28]. Thus, although *κ_e_* increased due to *n* optimization, the total thermal conductivity dropped by reducing *κ*_L_ and suppressing *κ*_b_. These results indicated iodine dopants synergistically regulated the electrical and thermal transport properties of Bi_2_SeS_2_.

**Figure 6:**
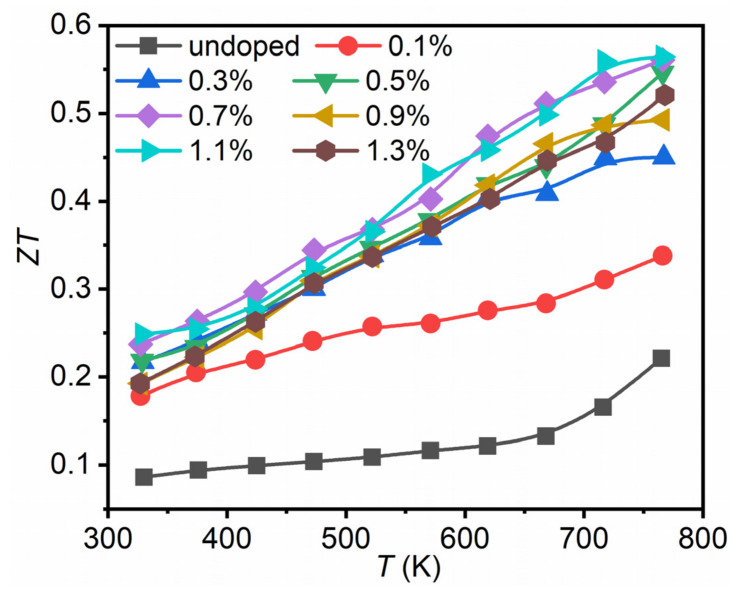
Dimensionless figure of merit (*ZT*) for Bi_2_Se_1−x_I_x_S_2_ (x = 0–1.3%).

Figure 6 shows the *ZT* values for Bi_2_Se_1−x_I_x_S_2_ (x = 0–1.3%). Combining with the optimized power factor and reduced thermal conductivity, a maximum *ZT* value of 0.56 at 773 K was obtained for Bi_2_Se_1−x_I_x_S_2_ (x = 1.1%), nearly three times higher than undoped Bi_2_SeS_2_, and indicating that iodine is an effective n-type dopant for Bi_2_SeS_2_. However, compared with Br doped samples [28], the present *ZT* value was much lower due to the degraded power factor at high temperatures. Further efforts can be devoted to the enhancement of the electrical transport properties of Bi_2_SeS_2_ at high temperature, such as dual-doping.

## 4. Conclusions

A series of Bi_2_Se_1−x_I_x_S_2_ (x = 0–1.3%) samples were prepared by combining a solid state reaction with SPS. Those results revealed that iodine is an effective n-type dopant for Bi_2_SeS_2_. The electrical conductivity increased due to optimized carrier concentration produced by the carrier provided defect *I_se_*. Combined with a moderate Seebeck coefficient (>150 µVK^−1^) due to its large effective mass, a high power factor of 697 μWm^−1^K^−2^ at 300 K was obtained. In addition, the total thermal conductivity decreased, primarily due to the decreased lattice thermal conductivity resulting from the enhanced phonon scattering. Thus, a maximum *ZT* of 0.56 at 773 K was achieved for Bi_2_Se_1−x_I_x_S_2_ (x = 1.1%), nearly three times higher than the pristine sample.

## Data Availability

The data presented in this study are available on request from the corresponding author.
